# Recognition, management, and patient perspectives of impulsive-compulsive disorders in Parkinson’s disease

**DOI:** 10.1177/1877718X251323922

**Published:** 2025-03-16

**Authors:** Mirjam Wolfschlag, Gustav Cedergren Weber, Jonathan Timpka, Daniel Weintraub, Per Odin, Anders Håkansson

**Affiliations:** 1 Clinical Addiction Research Unit, Department of Clinical Sciences Lund, Psychiatry, Lund University, Faculty of Medicine, Lund, Sweden; 2 Malmö Addiction Center, Department of Psychiatry Malmö-Trelleborg, Region Skåne, Kristianstad, Sweden; 3 Division of Neurology, Department of Clinical Sciences Lund, Lund University, Lund, Sweden; 4 Department of Neurology, Rehabilitation Medicine, Memory and Geriatrics, Skåne University Hospital, Lund, Sweden.; 5 Departments of Psychiatry and Neurology, University of Pennsylvania School of Medicine, Philadelphia, PA, USA

**Keywords:** Parkinson’s disease, disruptive, impulse control, and conduct disorders, surveys and questionnaires, medical records, delivery of health care, drug-related side effects and adverse reactions, dopamine agents, comorbidity, quality of life, deep brain stimulation

## Abstract

**Background:**

Impulsive-compulsive disorders (ICDs) are commonly acknowledged as side effects of dopaminergic therapy in Parkinson’s disease (PD). While many large-scale studies have focused on prevalences and high-risk treatments, little is known about practical management of ICDs in clinical care and patients’ experiences.

**Objective:**

To investigate how ICDs are recognized in clinical PD care, clinical features of patients with ICDs, and how patients are impacted by their ICD.

**Methods:**

Questionnaires were sent to all patients who reported ICD symptoms in the Swedish quality register for PD in Skåne County (n = 170) and patients’ medical records were screened for mention of ICDs. Core subjects were communication between clinician and patient, course and management of ICDs, and impact on different life domains.

**Results:**

Despite sufficient awareness of the ICD risk during PD treatment, there was limited communication between clinical care staff and patients regarding ICDs. Only 49% of patients had reported their ICD as part of clinical care, and only 14% had been asked about it. Additionally, collaboration with psychiatry was rare (12%). ICD severity increased over time with ongoing PD treatment, and most patients reported a mild to moderate impact of their ICD on close relationships, family, mental and physical health.

**Conclusions:**

This study identified insufficient communication about ICDs as part of clinical care in PD and a very limited involvement of mental health services. Thus, to improve prevention and treatment, ICDs should be recognized, monitored and treated more systematically in routine clinical care, and collaboration with mental health services should be increased.

## Plain language summary

Persons with Parkinson’s disease can develop impulsive and compulsive behaviors such as problem gambling or compulsive sexuality as a side effect of their treatment. Previous research has mostly focused on how to manage these side effects. However, little is known about clinical care professionals’ awareness and patients’ experiences. Furthermore, we lack knowledge about how these side effects develop over time and how they impact patients’ quality of life. To explore these issues, patients with Parkinson’s disease were selected from a medical register and had their medical records screened for impulsive and compulsive behaviors. Participating patients also responded to a questionnaire regarding their individual experience of interacting with clinical care. It was found that only half of the patients reported their issues with reduced impulse control to their clinical care providers. Merely 14% of patients were proactively asked about these symptoms by professional clinical care staff. In addition, few patients were referred to psychiatric care. Compulsive sexuality was the most common behavior, and almost all impulsive and compulsive behaviors got worse during the course of the treatment. The impact of impulsive and compulsive symptoms on patients’ quality of life was generally mild to moderate, but certain domains such as close relationships and mental health were affected severely in some patients. These findings point out gaps in the communication and management of impulsive and compulsive behaviors in patients with Parkinson’s disease. This could be improved by clinical care staff asking for these symptoms proactively and an increase in collaboration between routine care and mental health services.

## Introduction

Parkinson’s disease (PD) is a neurodegenerative disorder marked by both motor and non-motor symptoms.^1–3^ The cardinal symptoms are tremor, bradykinesia, rigidity and postural instability. Non-motor symptoms include autonomic dysfunction and psychiatric disturbances.

Since dopamine is a crucial neurotransmitter for both motor functions and reward-based learning, its manipulation through medications can impair impulse control.^4^ There is ample evidence that dopaminergic therapies elevate the risk for impulsive-compulsive disorders (ICDs), like compulsive sexuality, shopping or eating and problem gambling.^5^ Additionally, the unconscious repetition of simple motor patterns, called punding, and devoting excessive amounts of time to a regular activity done for enjoyment, called hobbyism, can occur during PD treatment.^6–10^ ICDs significantly reduce the quality of life for patients with PD,^11^ making it crucial to better understand their causes and management.^12,13^ Although ICDs are increasingly recognized as a side effect of PD therapy, many details remain unknown, particularly regarding long-term clinical care management.^12–14^ To date, there have been no comprehensive studies combining questionnaire data with register-based studies to explore the longitudinal course of ICDs, covering symptomatology, clinical care awareness and medication dosing.

We aimed to fill this gap by contributing more detailed insights into the progression of ICDs in PD. By examining the relationship between ICD development and individual therapies, our goal was to help identify better strategies to prevent ICDs in patients undergoing PD treatment. Our primary aim was to evaluate clinical care professionals’ awareness of ICDs, focusing on how effectively they identify and manage these disorders, as well as patients’ experiences with them. Additionally, we examined demographic and psychiatric risk factors, traced the progression of ICDs over time, and documented how the severity of these disorders impacts patients’ quality of life.

## Methods

### Study population

The study was approved by the Swedish Ethical Review Authority (diary number: 2022-06160-01). Patients were selected from the national Swedish quality registry for PD (ParkReg), a subdivision of the Swedish Neuro Registries.^15^ Screened were all subjects listed in Skåne County, an urban Swedish region with 1.4 million inhabitants, four larger cities and a wide coverage of university hospital care.^16^ Included were patients who had previously self-reported symptoms of ICDs (n = 172) in the extended Swedish version of the Non-Motor Symptoms Questionnaire (NMSQ)^17,18^ (additional question 31: “Have you experienced a behavioral change with increased gambling, sex, shopping, eating compared to before you developed PD?” or additional question 32: “Have you experienced an increase in other behaviors compared with before you developed PD, for example internet use, hobbies, artistic activities, writing or hoarding?”). Patients’ answers to the NMSQ had been collected in routine screenings for ParkReg.

### Medical records screening

The medical records analyzed were collected from Skåne University Hospital including in- or outpatient care received at the hospital but excluding primary care records. By reading through the records and complementing with search terms such as “impulse” or “gambling”, they were screened for patients who had ICDs mentioned in their medical records. Thereafter, additional information was extracted for all cases identified with an ICD. Included were the awareness of clinical care staff and patient of the risk for developing ICDs during the PD therapy and a possible adjustment of the therapy after the ICD was recognized. Moreover, the specific manifestation of an ICD, any contact with psychiatric or psychological care and any use of advanced PD therapies were extracted. Results were reported as absolute and relative frequencies.

### Questionnaire design and procedure

The questionnaire was designed specifically for this study combining several different research questions and was applied in Swedish. In the first part, patients were asked to rate their severity of six common manifestations of an ICD in PD patients (gambling, compulsive sexuality, compulsive buying, compulsive eating, hobbyism and punding), similar to the Questionnaire for Impulsive-Compulsive Disorders in Parkinson's Disease - Rating Scale (QUIP-RS).^19^ Four questions with severity scales from 0-4 resulted in a severity score of 0-16 for each behavior and of 0-96 in total. In addition to collecting information about the current state of the patients’ behavior at the time of answering the questionnaire, they were asked to rate their ICD retrospectively before the start of the PD therapy and six months after their therapy was initiated.

The next section of the questionnaire addressed the patients’ awareness of the risk to develop ICDs under PD medication and them noticing a possible connection of their ICD to their therapy. They were asked to report if they had mentioned their ICD in clinical care and how their therapy was adapted subsequently. Furthermore, patients were asked to select any advanced forms of therapy they had received and to describe its effect on their ICD.

In the last step, we asked for background information like other psychiatric or cognitive symptoms, substance use disorders and a family history of substance use disorders or ICDs. Patients were also asked to report if they had contacted psychiatric care for their ICD and to rate how severely different areas of their life, like close relationships or physical health, had been affected by their ICD. Results were reported as absolute and relative frequencies or underwent further statistical analysis. When calculating relative frequencies, missing values were excluded.

Questionnaires were sent to the patient’s home address and included a patient information sheet, a consent form and a response envelope. Patients were invited to seek help from relatives to answer the questionnaire if they wanted to and were compensated with a 100 SEK gift card for participation. Incoming response letters were handled by the study nurse and the digitalization and analysis of returned questionnaires was performed by the researchers without access to any personal data.

### Statistical analysis

IBM SPSS Statistics (version 29.0.0.0) was used to prepare and analyze data, GraphPad Prism (version 10.2.2) to analyze data and create graphs. The level of significance was set at α=0.05 and confidence intervals (CIs) were reported at 95%. For each patient, the individual increase in the self-reported severity score under PD therapy was calculated first for each behavior and then in total. If there was an increase both after six months of therapy and in the current state today compared to before the therapy, the higher value was used. For the total increase, the sum of the increase for all six behaviors was calculated.

The increase in the total severity score was compared between patients who had contacted psychiatric care for their ICD and those who had not, using a Mann-Whitney U test. Dependency of the increase in the total severity score on age and sex was analyzed by a linear regression model including both factors.

Changes in the self-reported severity score of different ICDs were compared within one behavior between all three timepoints (Before treatment, After 6 months, Today) using a mixed-effect model followed by Tukey’s multiple comparison test. Patients who did not report the specific behavior at any time point were excluded from that group, resulting in different patient numbers for each behavior.

## Results

### Included individuals for questionnaire and medical records analysis

**Figure 1.**
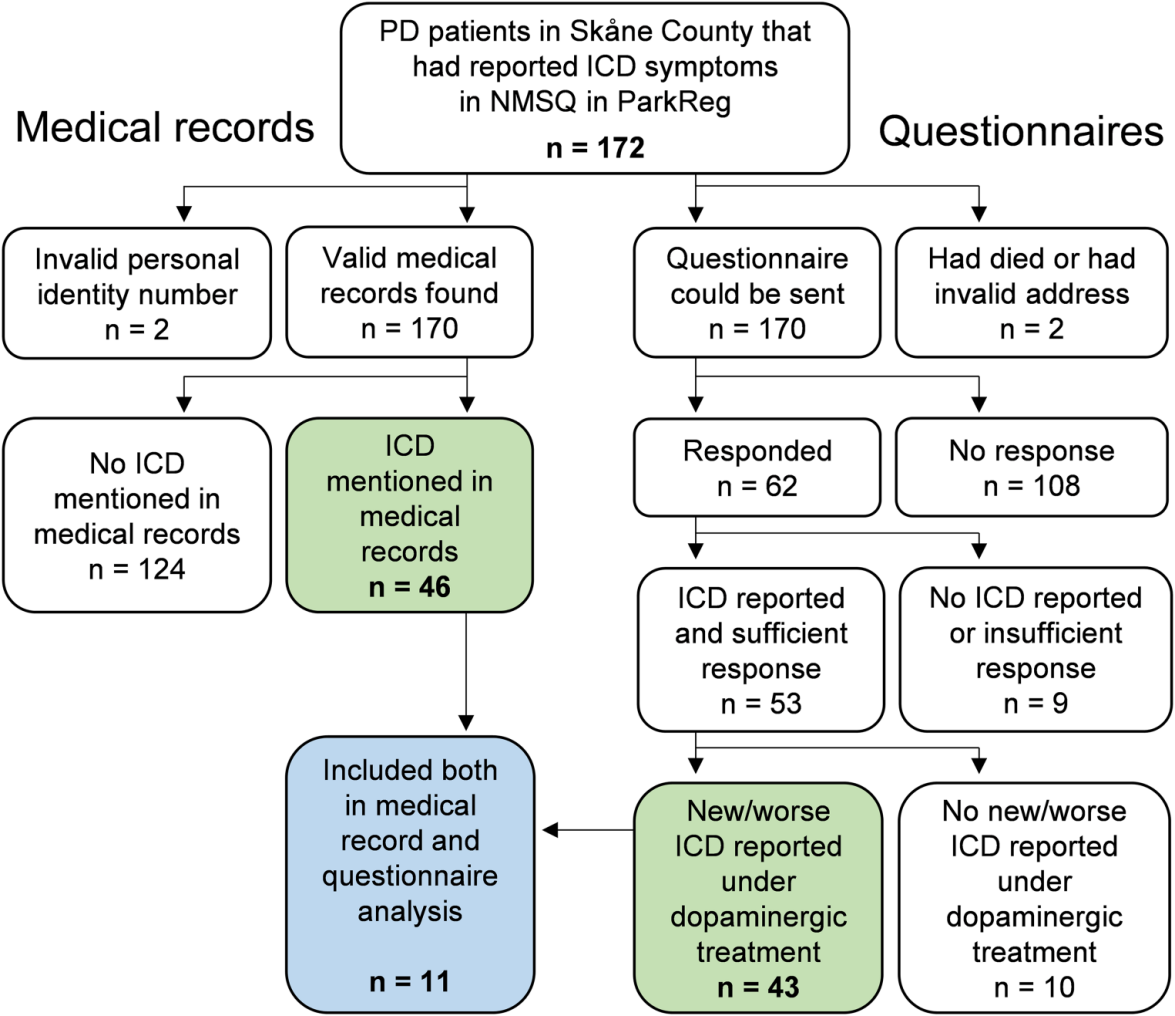
Flow diagram of included and excluded patients in the medical records and the questionnaire analysis. PD: Parkinson’s disease, ICD: impulsive-compulsive disorder, NMSQ: non-motor symptoms questionnaire, ParkReg: national Swedish quality registry for PD.

Of the 172 patients primarily identified with ICDs, two had to be excluded from the medical records screening due to an invalid personal identity number in the register (Figure 1). An additional 124 patients had no records of ICDs in their journals, eventually resulting in 46 studied individuals. Questionnaires were sent out to 170 patients, excluding two individuals who were deceased or had an invalid address (Figure 1). Of the 62 responses, nine were excluded based on an insufficient number of answers or the lack of any ICD mentioned. Another ten reported no new or worsened ICDs under PD treatment compared to before, resulting in 43 questionnaires included in the analyses. Eleven patients were included both in the medical records and the questionnaire study, 35 only in the medical records screening and 32 only in the questionnaire analysis.

### Recognition and management of impulsive-compulsive disorders in clinical Parkinson’s care

In 96% of all screened medical records (44/46), the PD treatment was mentioned explicitly as the likely cause for symptoms of impaired impulse control. As a response to ICDs, different types of therapy adjustments were made (Table 1). In two thirds of all patients, the medication suspected for causing the ICD was reduced in dose (44%) or discontinued (22%). The majority of adjustments were made to dopamine agonist treatment. Patients reported own awareness of ICDs induced by PD therapy in 72% of all cases (31/43). About half had noticed a relation between their changes in behavior and their medication themselves, found both in questionnaire answers (56%, 24/43) and medical records (46%, 21/46).

**Table 1.** Adjustments of Parkinson’s treatment in response to impulsive-compulsive disorders according to medical records. n = 46.

Adjustment		Frequencyabsolute (relative)
Medication discontinued		10 (22%)
	Dopamine agonist	7 (15%)
	Other	3 (7%)
Reduced dosage		20 (44%)
	Dopamine agonist	18 (39%)
	Other	2 (4%)
Change from immediate release to depot administration		1 (2%)
	Dopamine agonist	1 (2%)
	Other	0 (0%)
No adjustment		15 (33%)
	Dopamine agonists acknowledged as high-risk medication	6 (13%)
	No caveats with any medication mentioned	9 (20%)

**Figure 2.**
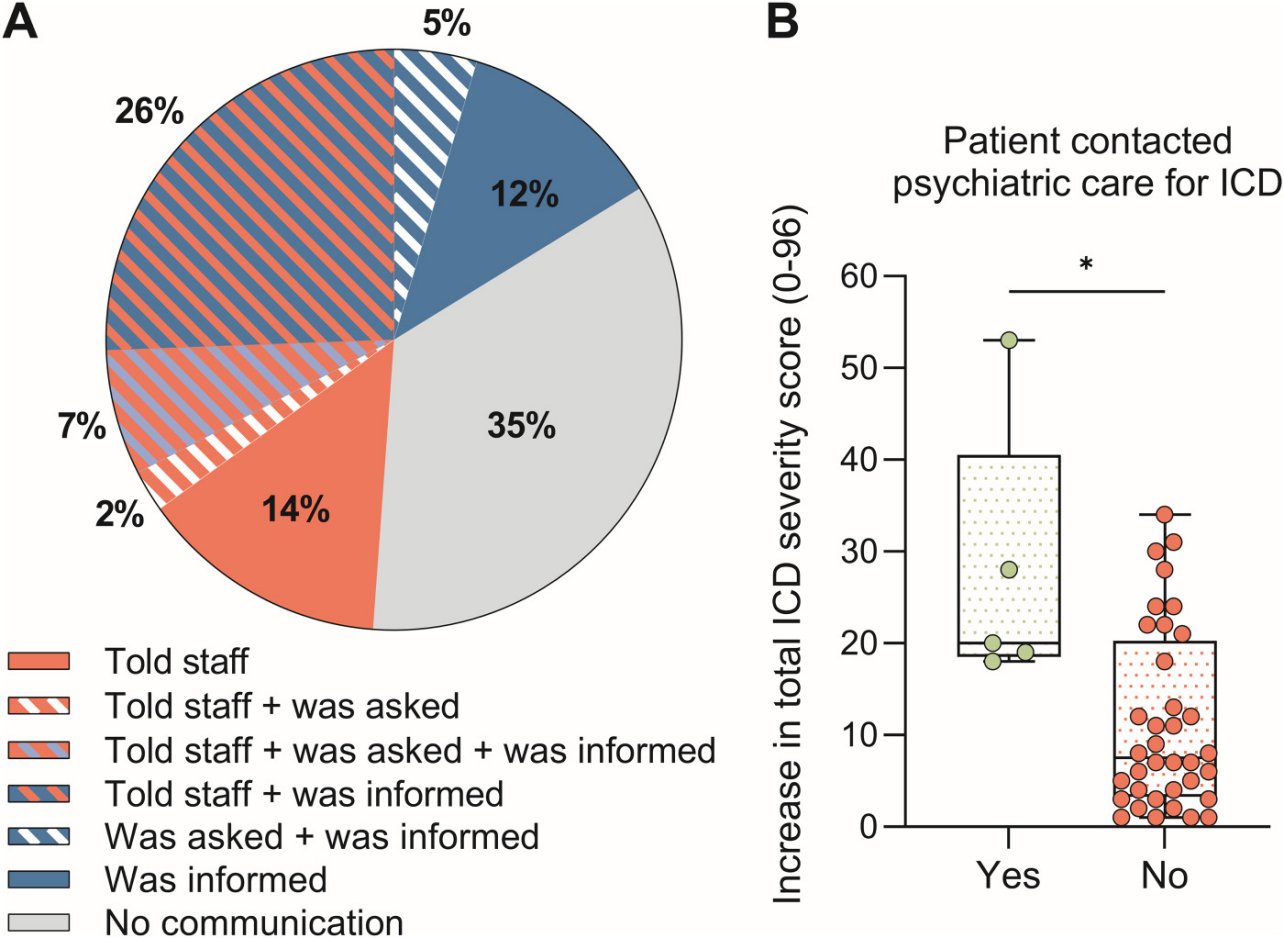
Involvement of clinical Parkinson’s disease and psychiatric care in addressing impulsive-compulsive disorders under Parkinson’s treatment. Based on patient reports, n = 43. (A) Overlapping relative frequencies of three types of staff-patient communication about impulse-compulsive disorders (ICDs) in Parkinson’s disease care. Fractions including red: Patient told the staff about their ICD (“Told staff”, n = 21). Fractions including blue: Patient was informed by staff about an increased ICD risk under Parkinson’s therapy (“Was informed”, n = 21). Fractions including white and light blue: Patient was asked actively by staff about experiencing ICDs under Parkinson’s therapy (“Was asked”, n = 6). Grey fraction: No communication between patient and staff took place regarding ICDs (“No communication”, n = 15). (B) Increase in self-reported total severity of ICDs during Parkinson’s therapy in patients who contacted psychiatric care for their ICD and those who did not. Group difference analyzed with Mann-Whitney U test, U = 31, p = 0.016.

About half of the patients that reported an increase of ICDs during their PD therapy in the questionnaire had mentioned this issue to their clinical care staff (Figure 2A). Fourteen percent had been actively asked about experiencing any ICD during appointments. In 33% of all cases, the patient was informed about the risk of developing an ICD at the start of the therapy, with an additional 16% being informed later on during the therapy. Taken together, no form of communication about ICDs had taken place during clinical care visits in 35% of all cases.

In most cases, ICDs were handled exclusively within clinical PD care according to patient reports. Only a few patients with severe behavioral symptoms and multiple ICDs had been in contact with psychiatric care, either referred by their PD care or on own initiative (Figure 2B). Compared by a Mann-Whitney U test, self-rated ICD severity was a significant factor for seeking psychiatric care (U = 31, p = 0.016). The ICD course during PD therapy was similar for patients with and without contact to psychiatric care. Furthermore, none of the patients with screened medical records had received a specific diagnosis for their ICD. Thirteen percent (6/46) had contacted psychiatric care for their ICD, while an additional 30% (14/46) had sought psychiatric care for other mental health reasons primarily.

### Frequencies and development of different impulsive-compulsive disorders during Parkinson’s therapy

**Figure 3.**
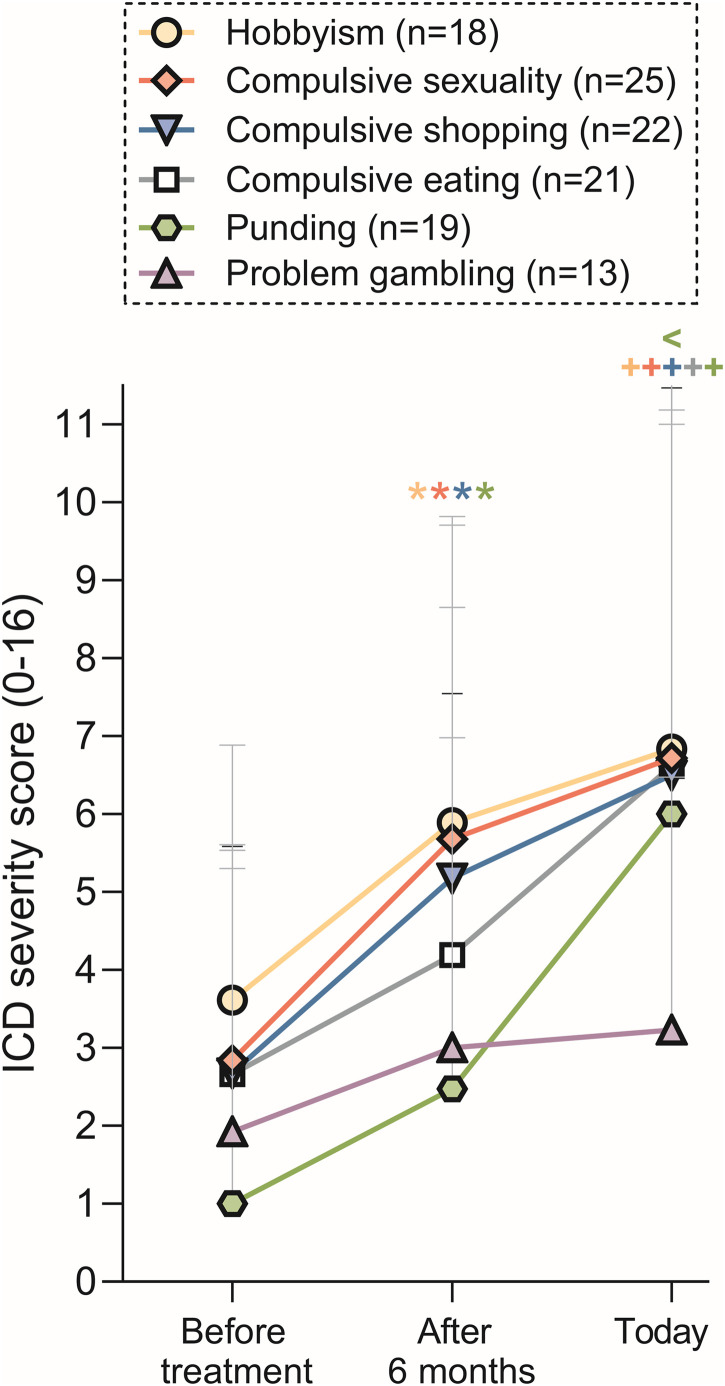
Self-reported severity score of six different impulsive-compulsive disorders before the Parkinson’s therapy, six months after therapy initiation and in current state today. n(total) = 43, patients who did not report the specific behavior at any time point were excluded from that group (see different amount of n in legend). Values are shown as mean + standard deviation and were compared within one behavior between the three timepoints using a mixed-effect model and Tukey’s multiple comparison test: *p < 0.05 for After 6 months vs. Before treatment, ^+^p < 0.05 for Today vs. Before treatment, ^<^p < 0.05 for Today vs. After 6 months.

Compulsive sexuality was the most commonly self-reported ICD, followed by compulsive shopping, compulsive eating, punding, hobbyism and problem gambling (Figure 3). Analyzing medical records in comparison, 28% were described with compulsive sexuality (13/46), 24% with problem gambling (11/46), 20% with compulsive shopping (9/46), 13% with compulsive eating (6/46), 2% with hobbyism (1/46) and 33% without mentioning any specific ICD (15/46). Moreover, 67% of all patients (29/43) and 17% of the medical records (8/46) reported more than one ICD.

All ICDs increased mildly in severity over time after the PD treatment had started, not considering if the therapy had been adapted after the ICD or not (Figure 3). Self-reported ICD symptom severity was significantly higher after six months of PD treatment compared with before the therapy for hobbyism, compulsive sexuality, compulsive shopping and punding. Furthermore, the severity of all ICDs but problem gambling had increased significantly from before the therapy, up until the current state at data collection. Punding severity was additionally increased at data collection in comparison to after six months of PD therapy. On average, all ICDs were present to a mild degree before the therapy with highest severity ratings for hobbyism, compulsive sexuality, shopping and eating. The individual course of ICDs differed a lot between patients, but no general differences between patients reporting ICD symptoms present prior to their PD therapy and ones reporting ICD symptoms only after the therapy had started could be detected.

### Clinical aspects and consequences of impulsive-compulsive disorders during Parkinson’s therapy

In the patient group whose medical records were analyzed, the mean age was 65.9 ± 11.1 years and 34 patients (74%) were male. In comparison, the mean age for patients included in the questionnaire analysis was 69.3 ± 11.9 years and 31 patients (72%) were male. The self-rated ICD severity in the questionnaire was not influenced by the patient’s age or sex, confirmed by a linear regression (n = 43; β_age_= -0.138, p = 0.368; β_male sex_= 0.123, p = 0.975).

Regarding psychiatric and cognitive comorbidities in patients who reported ICD symptoms, highest frequencies were observed for sleep disturbances, lack of motivation and vivid dreams (Table 2). About a third of all patients with ICDs each reported symptoms of depression, anxiety, mental exhaustion and hallucinations. Alcohol use disorder and other substance use disorders were less common and a fourth of all patients who presented ICD symptoms also reported a family history of alcohol use disorder.

**Table 2.** Self-reported psychiatric and cognitive comorbidities and family history of Parkinson’s disease patients with impulsive-compulsive disorders.

Comorbidity	Frequency absolute (relative)	n
Depression	13 (30%)	43
Anxiety	13 (30%)	43
Mental exhaustion	14 (33%)	43
Lack of motivation	24 (56%)	43
Sleep disturbance	27 (63%)	43
Hallucinations	14 (33%)	43
Vivid dreams	24 (56%)	43
Alcohol use disorder	4 (10%)	41
Other substance use disorder than alcohol	1 (2%)	41
Other	3 (7%)	43
		
*Family history*		
Impulsive-compulsive disorder	7 (18%)	39
Alcohol use disorder	10 (25%)	40
Other substance use disorder than alcohol	3 (8%)	40

According to the screened medical records, 43% of all patients had received deep brain stimulation (DBS) treatment (20/46), while DBS treatment was less likely to be self-reported (22%, 9/41). No reliable information about any pharmacotherapy via pump administration could be extracted from the medical records. In the questionnaire, one patient reported pump therapy with apomorphine (2%) and one with levodopa-carbidopa intestinal gel (2%). Two thirds of the patients that initiated DBS treatment had experienced an improvement of their ICD after the procedure (67%, 6/9) and none reported a worsening of the ICD.

**Figure 4.**
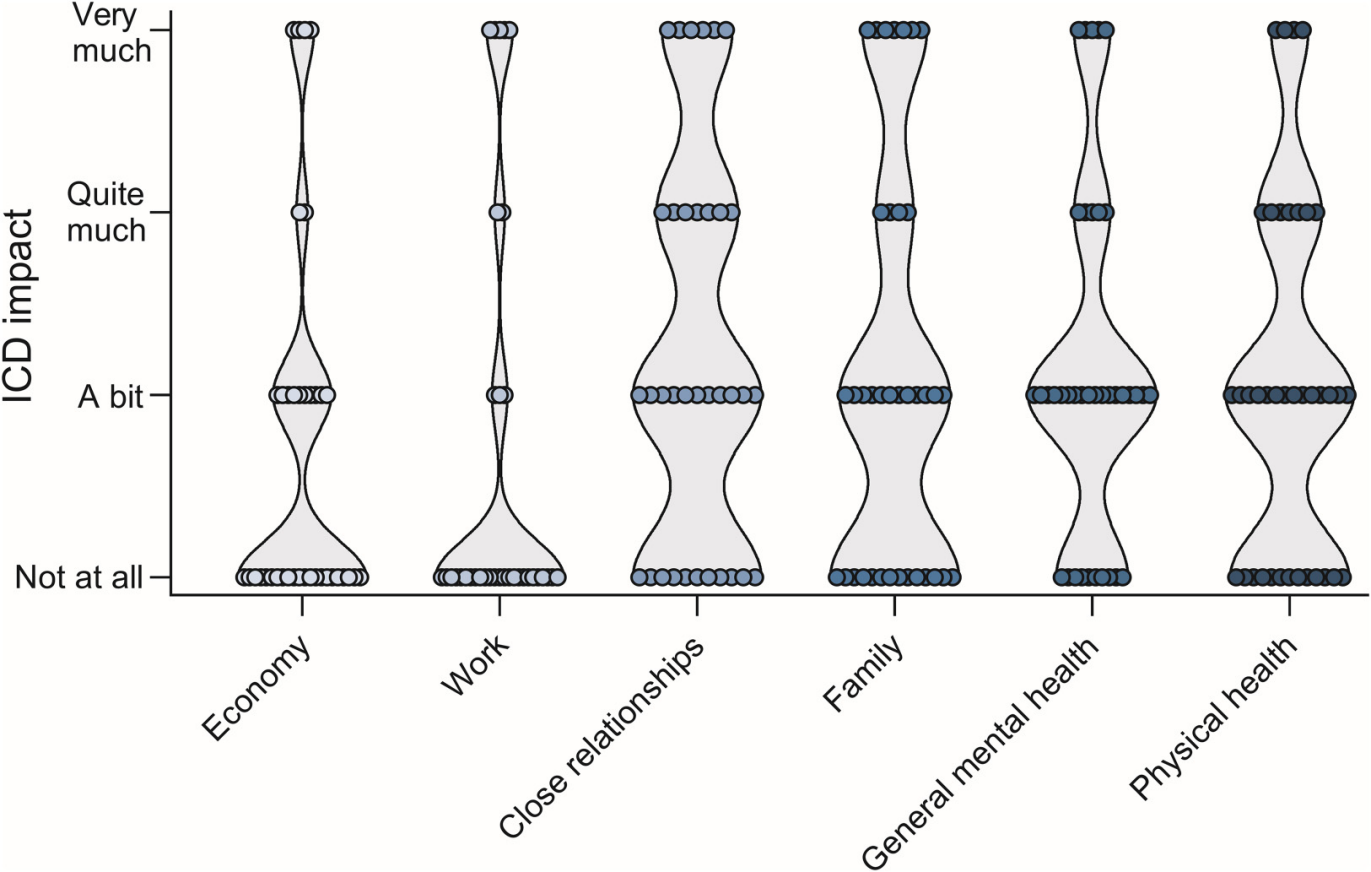
Self-reported impact severity of impulsive-compulsive disorders on different areas in patients’ lives. n(Economy) = 38, n(Work) = 35, n(Close relationships) = 35, n(Family) = 37, n(General mental health) = 38, n(Physical health) = 38.

Most patients reported a mild effect of their ICD on their general mental health, while close relationships and family life were affected mildly to moderately, with a few patients rating the ICD impact as very high (Figure 4). The impact on physical health was similar, with mild to moderate self-ratings in most cases. Patients’ economy and their work, if applicable, were barely affected by their ICD.

## Discussion

This study provides significant insights into the recognition, management and consequences of ICDs among patients receiving PD therapy. By analyzing both medical records and patient-reported data, the findings shed light on the limitations in current clinical care practices related to ICDs in PD, as well as the challenges patients face in managing these behaviors. The limited clinical care response and the lack of communication between professionals and patients regarding ICD risk underscore the need for improved care strategies.

One of the key findings was the high awareness of ICDs among clinical care professionals, with 96% of medical records acknowledging PD medication as the likely cause for impaired impulse control. However, the actual management of these disorders was less consistent. While reducing or stopping dopamine agonists is known to improve ICD symptoms, adjusting other dopaminergic drugs like levodopa shows no effect on these issues.^20–22^ In only 22% of cases was a dopamine agonist discontinued, and in 44% of cases, the dosage was reduced. These findings are consistent with prior studies, which also highlight that although awareness of ICD risk among clinical care providers is improving, significant gaps remain in proactive management.^6^ It appears that a major barrier to effective intervention is the limited integration of psychiatric consultation into the treatment of PD patients experiencing ICDs. This is particularly concerning given that 88% of ICDs were managed solely within PD care, with only a minority of patients being referred to psychiatric care.^23^ Seeking psychiatric care was associated with more severe ICDs. Patients with less severe ICDs might have been treated by specialists outside psychiatry, but the finding could also reflect neglect of milder symptoms by either the patient, the physician, or both. Alternatively, the symptoms might be subsyndromal, thus not necessitating treatment. Interestingly, the ICD course compared between patients with milder symptoms but without contact to psychiatric care and patients with severe ICDs under psychiatric treatment was in general similar. This could indicate that the support by psychiatric care compensated for more severe symptoms and that most patients would benefit from psychiatric care involvement when handling their ICD. The disconnect between PD care and psychiatric services observed here mirrors previous findings in the literature.^7^ While dopaminergic therapies are crucial for managing motor symptoms, they simultaneously increase the risk of ICDs, necessitating a multidisciplinary approach that integrates neurology and psychiatry to ensure both motor and behavioral symptoms are adequately addressed.^24^ The lack of psychiatric diagnoses and limited use of psychiatric care indicate a systemic issue that could be mitigated by better cross-disciplinary collaboration.^6^

Our findings also indicate substantial gaps in communication between patients and caregivers. Although most patients were aware of ICDs and many recognized their behaviors as linked to their medication, only 49% reported these issues to their clinical care providers. Furthermore, merely 14% of patients were proactively asked about ICD symptoms by care professionals. These statistics suggest a lack of systematic inquiry into ICDs during patient visits, which is consistent with previous studies that reported similar issues in provider-patient communication.^9^ Proactive screening and regular discussion of ICD risk is crucial, as it has been shown to lead to better management and outcomes for PD patients.^8^ Improving this communication is vital, as proactive discussion of ICDs could lead to earlier identification and management of these debilitating symptoms,^12,25^ potentially preventing significant deterioration in quality of life.

Interestingly, the study found that patients who had undergone DBS reported improvements in ICD symptoms, with two-thirds noting symptom alleviation post-DBS. This aligns with previous research suggesting that DBS may have beneficial effects on ICDs in certain patients.^26,27^ Other studies have shown mixed outcomes.^28^ DBS in most cases leads to significantly reduced dopaminergic medication,^29^ but the exact neuromodulation of DBS itself is currently not well understood.^20,30,31^ It has been speculated that potential reduction in impulsive behaviors following DBS may be partially due to the modulation of dopamine signaling pathways implicated in reward processing and impulse control.^32^ While this study presents limited data on pump therapy, partially due to unreliable data from the medical records, there is growing evidence that treatment with levodopa-carbidopa intestinal gel poses a low risk of causing ICD and shows promising potential for improving pre-existing ICD.^33,34^ In contrast, apomorphine infusion pump therapy appears to carry a risk of developing ICD, though conflicting results have been reported.^13,35–38^ These findings highlight the potential role of advanced therapies not only in motor symptom management but also in addressing non-motor complications like ICDs. However, the variation between self-reported and medical record-documented rates of DBS suggests a potential underreporting or miscommunication issue regarding treatment outcomes that needs further exploration.^39^

The detailed analysis of different ICDs revealed that compulsive sexuality, shopping and eating were the most frequently reported behaviors, consistent with prior studies that have identified these as common manifestations of ICDs in PD.^8^ Patients with all subtypes of ICDs reported symptoms already before initiation of PD therapy. A pre-PD history of ICDs is a known risk factor for developing ICDs during PD,^40^ highlighting the need for preemptive screening. Importantly, all ICDs, except for problem gambling, showed significant increases in severity after six months of PD treatment. This reinforces the chronic and progressive nature of ICDs under dopaminergic therapy, emphasizing the need for ongoing monitoring rather than a one-time assessment.^11^

In line with the literature, psychiatric comorbidities were common,^41^ with a third of patients with ICDs reporting symptoms of depression, anxiety, mental exhaustion and hallucinations respectively. The most frequent comorbidities associated with ICDs in this study were sleep disturbance, vivid dreams and lack of motivation. Vivid dreams might represent REM sleep behavior disorders (RBD).^42^ These manifestations may help identify and monitor patients at risk for ICDs. Sleep disorders, particularly RBD, are associated with worse PD and more severe neuropsychiatric symptoms,^42^ possibly indicating broader non-motor frailty. Furthermore, RBD and ICDs have been shown to exhibit high comorbidity.^43^ Alcohol use disorder was reported in less than one in ten patients and other substance use disorders in less than one in 20 patients. These findings align with the theory of different mechanisms behind ICDs and substance addiction.^44–46^ However, one in four patients reported family history of alcohol use disorder and one in six patients reported family history of ICDs, suggesting some hereditary component^47,48^ and/or effect of intergenerational trauma.^49,50^

The impact of ICDs on different areas of patients’ lives was generally mild to moderate, but certain domains such as close relationships and mental health were affected severely in some patients. Interestingly, patients’ physical health seemed almost equally affected, pointing out patients who report ICD symptoms as a possible risk group for somatic comorbidities. Previous research has shown that ICDs can significantly reduce life satisfaction and increase caregiver burden.^11^ Addressing these behaviors is thus not only essential for the patients’ quality of life but also for reducing stress on caregivers and family members.^24^

A strength of this study is the approach of validating patients’ self-reports additionally through medical records authored by clinical care staff. We expect a higher reliability of our results when applying this complementary design compared to only using patient questionnaires or medical records separately. However, only a minority of participants were included in both studies, limiting this comparative advantage. Participants were preselected by screening for patients who had previously reported symptoms of ICDs. Thus, we assume a study population of high relevance to our research questions. Both groups of patients, included in the questionnaire and the medical record analysis, were characterized by a representative age and sex distribution for a PD population.^3,9^

However, this study has several limitations as well. The sample size was small and the inclusion of patients in a single region limit the generalizability of the findings. Furthermore, relying on patient self-reports introduced a potential recall bias, particularly regarding retrospective severity ratings of ICDs. Since individual timelines were not collected, it was impossible to determine the interval between six months post-therapy initiation and the present. Future research should therefore consider longitudinal follow-up with a larger and more diverse patient cohort to validate these findings. Additionally, interventions designed to bridge the gap between PD and psychiatric care should be explored, as well as systematic screening procedures that could help identify ICDs both before and during PD treatment.^32^

## Conclusion

The findings from this study underline significant gaps in the management of ICDs in PD, including limited communication about ICD risk and insufficient psychiatric care involvement. Enhancing multidisciplinary care approaches, particularly integrating psychiatric expertise into PD management, could lead to better outcomes for patients suffering from ICDs. Moreover, proactive communication and systematic inquiry into ICDs during routine care are crucial steps toward improving both the identification and management of these complex behaviors.
